# Cost-effectiveness analysis of intensive and emerging rehabilitation therapies in children with cerebral palsy: an observational cohort study using real-world evidence and microsimulation modelling

**DOI:** 10.1186/s13561-026-00768-2

**Published:** 2026-05-01

**Authors:** Diana Marcela Nova-Díaz, Sergio Aguilera-Albesa, Eduardo Sánchez-Iriso

**Affiliations:** 1 Department of Economics, Public University of Navarra, Pamplona, Spain; 2 Paediatric Neurology Research Group, Navarrabiomed, Pamplona, Spain; 3 University Hospital of Navarra, Pamplona, Spain

**Keywords:** Cerebral palsy, Cost-effectiveness analysis, Real-world evidence, Microsimulation modelling, Pediatric rehabilitation, Health technology assessment

## Abstract

**Objective:**

To evaluate the short- and long-term cost-effectiveness of integrating Intensive and Emerging Rehabilitation Therapies into standard care for children with cerebral palsy (CP), compared to standard care alone, through a hybrid approach combining prospective real-world data analysis and individual-level microsimulation over a lifetime horizon.

**Methods:**

A prospective observational cohort of 148 children with CP, stratified by Gross Motor Function Classification System levels, was followed over 12 months. Short-term incremental costs and quality-adjusted life years (QALYs) were estimated using seemingly unrelated regression equations (SURE) based on EQ-5D-Y scores. Costs were assessed from the Spanish public healthcare system perspective. To extrapolate long-term outcomes, an individual-level microsimulation model projected costs and QALYs over a 30-year horizon, applying a 3% annual discount rate.

**Results:**

Compared with standard treatment, Therasuit and intensive physiotherapy demonstrated the most favourable cost-effectiveness profiles. Therasuit generated 0.222 additional QALYs at an incremental cost-effectiveness ratio (ICER) of €18,830/QALY, while intensive physiotherapy generated 0.216 additional QALYs at €31,772/QALY. Other therapies, including occupational therapy and hippotherapy, were dominated by standard care. Long-term microsimulation provided additional insights beyond short-term findings by capturing delayed benefits, which in some cases led to different cost-effectiveness rankings among therapies. Therasuit produced 5.49 additional QALYs at an ICER of €12,922/QALY compared to standard care, and intensive physiotherapy produced 4.92 additional QALYs at €25,789/QALY. Homeopathy and the Petö Method were cost-effective under broader willingness-to-pay thresholds but were less efficient.

**Conclusions:**

Therasuit and intensive physiotherapy are high-value options when added to standard care for children with CP. Findings support prioritising Intensive and Emerging Rehabilitation Therapies using real-world evidence and modelling to guide sustainable healthcare decision-making.

**Supplementary Information:**

The online version contains supplementary material available at 10.1186/s13561-026-00768-2.

## Introduction

Cerebral palsy (CP) is the most common cause of motor disability in childhood, with a global incidence ranging from 1.4 to 3.0 per 1,000 live births [1]. It is a lifelong neurological disorder characterized by impairments in muscle tone, strength, sensation, and coordination, which compromise both unimanual and bimanual functionality. Consequently, children with CP experience significant functional difficulties in essential daily activities such as eating, drinking, walking, grasping, reaching, releasing, and manipulating objects [2]. These limitations not only affect self-care, school participation, and home activities but also have a profound impact on health-related quality of life (HRQoL) [3–5].

Among the different motor impairments in CP, spasticity is the most prevalent, affecting between 70% and 91% of children [6, 7]. This condition leads to substantial functional limitations and secondary complications such as pain, sleep disturbances, and pressure sores. In response to these challenges, motor therapies play a crucial role in improving HRQoL by enhancing physical, social, and emotional well-being [4, 8]. Over the past years, various therapeutic approaches have been developed to target mobility disorders in children with CP. In their systematic review, Novak et al. [9, 10] identified moderate to strong evidence supporting intensive movement therapy models and therapies based on neuromotor and sensory learning, such as hippotherapy, the Therasuit therapy and the Petö method [9].

Therapeutic interventions for individuals with cerebral palsy can be broadly categorized into standard treatment and *Intensive and Emerging Rehabilitation Therapies* [11]. Standard treatment includes conventional physical rehabilitation approaches aimed at preserving or improving motor function, communication, and activities of daily living [12, 13]. Within the broader category of intensive and emerging therapies, two subgroups can be identified. First, *Intensive Rehabilitation Therapies* refer to enhancements or extensions of standard care intended to optimize clinical outcomes. These include interventions such as physiotherapy, occupational therapy, and speech therapy [11]. Second, *Emerging Rehabilitation Therapies* comprise newer or less conventional interventions that have been gradually adopted in clinical practice. These are sometimes used alongside standard care and include hippotherapy, aquatic therapy, homeopathy, the Petö method, and therapeutic suits such as Therasuit [14]. While standard treatment remains the foundation of CP management, growing interest in these additional therapies reflects their perceived potential to improve mobility, coordination, and overall well-being. However, their broader implementation in routine practice requires solid evidence on both their therapeutic value and cost-effectiveness, underscoring the need for robust economic evaluations.

In the present study, the principal therapies evaluated encompass both intensive and emerging rehabilitation modalities. Physiotherapy and occupational therapy represent intensive, evidence-based approaches designed to improve postural control, strength, and functional independence through repetitive and goal-oriented exercises [13]. Speech therapy focuses on improving communication and feeding skills in children with oral-motor and swallowing impairments. Among emerging interventions, hippotherapy applies the rhythmic movement of the horse to stimulate neuromotor and sensory integration, improving trunk stability and balance [9]. The Petö method, or conductive education, integrates motor learning with cognitive and social development in a structured pedagogical framework. Therasuit therapy employs a soft dynamic orthotic suit combined with intensive exercise programs to promote motor alignment and functional re-education [9, 15]. Lastly, homeopathy was included as a non-conventional complementary intervention occasionally used in pediatric neurorehabilitation settings [11]. Collectively, these therapies illustrate the range of rehabilitation strategies currently implemented for children with CP and provide the empirical basis for their joint economic evaluation.

In recent years, systematic reviews have identified prevention strategies with strong evidence (e.g., antenatal corticosteroids, magnesium sulphate, caffeine, and hypothermia) as well as multiple allied health, medical, surgical, pharmacological, and regenerative interventions [14, 15]. Some interventions have transitioned from emerging to effective, such as botulinum toxin with casts, goal-directed training, and hippotherapy [9]. However, despite advancements in treatment, many interventions still lack robust evidence regarding their effectiveness, particularly from an economic perspective [11, 13]. This gap is critical, as assessing the cost-effectiveness of these therapies is essential for ensuring optimal resource allocation and long-term sustainability within public healthcare systems [11, 16].

Regardless of their potential benefits, CP therapies impose a substantial financial burden. In Spain, the annual cost per child with CP is estimated at €102,135 more than thirty times the average per capita healthcare expenditure. Additionally, medical costs for children with CP are approximately ten times higher than those for healthy children [15, 17]. Given these economic constraints, it is crucial to evaluate not only the clinical effectiveness of existing and emerging therapies but also their cost-effectiveness [13, 15, 18]. Transparent and rational decision-making processes for the reimbursement of new therapies are necessary to ensure sustainable healthcare spending.

Cost-effectiveness analysis (CEA) provide structured approaches to assess the value of healthcare interventions [12], quantifying outcomes in quality-adjusted life years (QALYs), facilitating comparisons across interventions and conditions. CEA are particularly valuable in chronic pediatric conditions like CP, where caregiver burden and long-term disability have economic implications beyond direct medical costs [19, 20]. In Spain, the generally accepted willingness-to-pay (WTP) threshold ranges from €25,000 to €60,000 per QALY gained, underscoring the need for interventions to demonstrate both clinical and economic value [21–23] The EQ-5D-Y is a widely used measure for assessing HRQoL in pediatric populations and is recommended for economic evaluations [8, 12, 24]. While its application in children with CP is still limited, the EQ-5D-Y represents an appropriate tool for health economic evaluations and contributes to generating comparable HRQoL data in this population [25].

Despite the increasing use of Intensive and Emerging Rehabilitation Therapies in clinical settings, prospective studies evaluating their cost-effectiveness in children with CP remain scarce. To address this gap, we conducted a CEA of integrating these therapies with standard care, using real-world data from a pediatric cohort in Spain and linking the 12-month observational follow-up to an individual-level microsimulation to project long-term outcomes. This microsimulation model is an individual-based analytic framework that simulates each child’s lifetime course of costs and health outcomes, using the observed 12-month results as input data. It enables extrapolation beyond the trial horizon and captures patient-level variability, providing a comprehensive estimation of long-term cost-effectiveness. The evaluation was conducted from the Spanish public healthcare system perspective, including direct medical costs, with a 3% annual discount rate for costs and QALYs over a 30-year horizon.

## Methods

### Study design and setting

This research forms part of the Navarra Pediatric Cerebral Palsy Study (EPCINA: https://www.navarrabiomed.es/es/investigacion/proyectos/gona-3524-estudio-de-paralisis-cerebral-edad-pediatrica-navarra-epcina), a prospective observational cohort conducted at the University Hospital of Navarra (HUN) in Spain. The design incorporated both retrospective data on healthcare resource use and costs, and prospective assessment of HRQoL over a 12-month follow-up period. The economic evaluation comprised a CEA, comparing standard treatment alone with standard treatment supplemented by *Intensive and Emerging Rehabilitation Therapies*. As this was an observational study, no interventions were allocated; participants were classified into treatment groups according to the care they were receiving, either within or outside the public healthcare system.

The analysis was conducted from the *perspective of the Spanish public healthcare system*, including all direct medical costs. Resource use and cost data were collected retrospectively for the 12 months prior to cohort entry, and prospectively throughout the follow-up. HRQoL was assessed prospectively using validated preference-based instruments. Incremental costs and effects of Intensive and Emerging Rehabilitation Therapies were estimated using data collected between July 2023 and December 2024, assuming *12 months of effective therapy in the base-case cost-effectiveness framework.*

This economic evaluation was designed and reported in accordance with the *Consolidated Health Economic Evaluation Reporting Standards 2022 (CHEERS 2022)* to ensure transparency, consistency, and methodological rigor throughout the study [26]. The CHEERS checklist provides a structured framework for reporting cost-effectiveness and cost-utility analyses and guided both the planning and preparation of this manuscript. In addition, this observational cohort study was designed and reported in line with the STROBE (Strengthening the Reporting of Observational Studies in Epidemiology) statement for cohort studies [27]. *The completed CHEERS 2022 and STROBE checklists are provided in the Supplementary Material* (Tables S3–S4).

### Study population

Children were eligible for inclusion if they met the following criteria: (1) a confirmed diagnosis of CP; (2) age between 3 and 18 years; (3) residency in Navarra; (4) informed parental consent and willingness to report healthcare resource use and HRQoL; and (5) current participation in therapeutic treatment, either within or outside the public healthcare system. Exclusion criteria included age under 3 years (due to the lack of definitive diagnosis at this stage) [28], unconfirmed diagnoses, or inability to complete required assessments.

The final sample comprised 148 children aged 3–18 years with a confirmed diagnosis of CP, representing 100% of the diagnosed and eligible pediatric CP population in Navarra according to the regional registry. Although the total estimated number of children with CP in the region is 201 (ages 0–18 years) [29], the analysis was restricted to the 3–18-year age range to comply with international diagnostic and surveillance standards (SCPE) and ensure comparability with other epidemiological studies. The sample included children across all Gross Motor Function Classification System (GMFCS) levels.

### Data collection, data sources, and costing approach

Cost and resource-use data were collected *prospectively and retrospectively* for each participant over a 12-month observation period using the *Economic Burden Questionnaire for Cerebral Palsy (EBQ-CP)* [17], a structured instrument composed of three sections.

*Part 1* was completed by the interviewer/researcher, who extracted administrative and clinical data from institutional sources (Navarra Health Services, University Hospital of Navarra, and collaborating centers). This section *captured resource use* related to *standard medical care* provided through the public healthcare system, including hospitalizations, specialist consultations, diagnostic tests, medications, and publicly funded rehabilitation services, as well as *government subsidies* or institutional aid. *Parts 2 and 3* were completed by the *primary caregiver* through a *semi-structured interview* administered by the same interviewer. *Part 2* collected information on *out-of-pocket expenses* related to *therapies and services outside the public health system* (Intensive and Emerging Rehabilitation therapies). *Part 3*, the *Cost Diary*, documented the *time units* the main caregiver devoted to accompanying the child to therapies, the *number of sessions*, and *frequency* of attendance, providing a detailed record of time and activity use.

Caregivers provided detailed information on healthcare resource use and costs through the EBQ-CP, including both out-of-pocket expenditures for Intensive and Emerging Rehabilitation Therapies (IERT) and use of public healthcare services (Standard Treatment). Data on IERT were obtained directly from caregivers, who reported the total cost paid in collaborating private or semi-subsidized centers. These costs were recorded as integral service prices, meaning that each IERT unit cost already included all components of the therapy (professional fees, materials, equipment, facilities, administrative expenses, and medical supervision). Therefore, no additional or itemized sub-costs were added. For public healthcare resources (Standard Treatment components such as hospital visits, diagnostic tests, medications, etc.), administrative and institutional records were used as the primary data source, while caregiver reports served to cross-check or complete missing information. When multiple data sources were available for the same resource (e.g., administrative record, caregiver report, or supporting document), administrative or documentary evidence was prioritized to minimize recall bias and ensure accuracy.

**Fig. 1:**
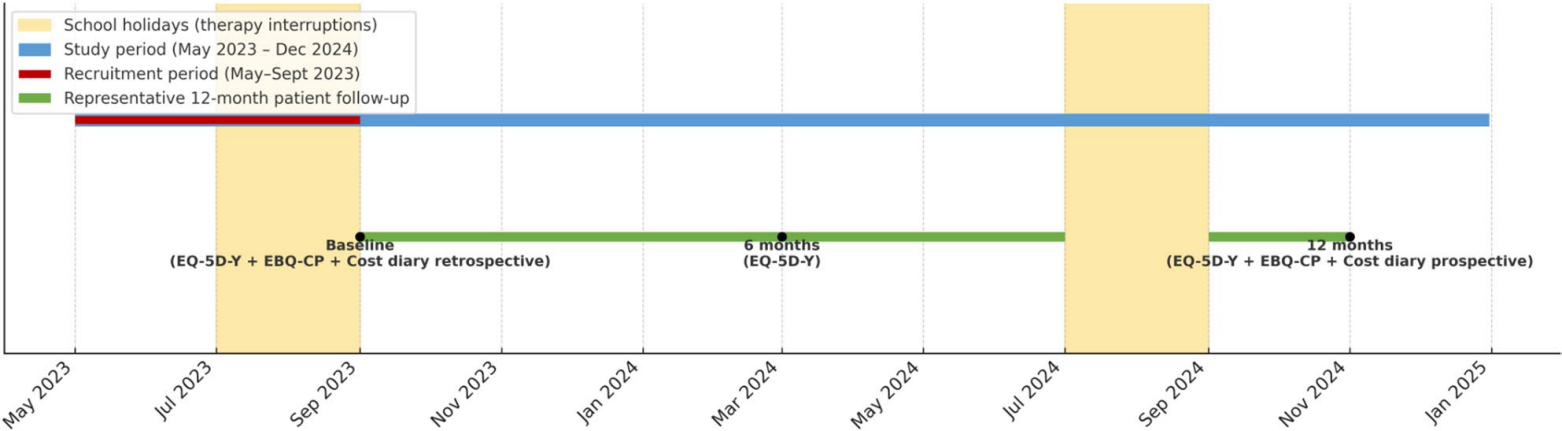
Study timeline: data collection schedule for EBQ-CP and EQ-5D-Y interviews. Notes: This figure represents the entire study period (May 2023 to December 2024) and the representative 12-month follow-up window (September 2023 to November 2024). The EQ-5D-Y interviews were conducted at three points in time: at baseline, at 6 months, and at 12 months. EBQ-CP were administered twice: retrospectively at the start of the study (covering the previous 12 months, with all retrospective data validated against institutional clinical and administrative records for all participants) and prospectively at the end of follow-up. The areas shaded in yellow indicate the school holiday months (July–September), during which therapy sessions were reduced or suspended. These periods were excluded from the treatment exposure analysis and cost-effectiveness analysis due to low therapeutic intensity

The EBQ-CP and the cost diary were administered at two time points: at study baseline, to retrospectively capture the previous 12 months, with all data validated against clinical records, institutional administrative records, and information provided by the primary caregiver regarding resource use for all participants’ treatments; and at study completion, to record real-time resource use and costs during follow-up. Standard treatment costs were fully covered by the public healthcare system, whereas additional expenses related to IERT were partially funded by families and partially subsidized by public or institutional sources. Additionally, three structured interviews were conducted to collect EQ-5D-Y data at study baseline, at 6 months, and at 12 months (Fig. 1).

### Valuation rules and data sources

For resources charged to the public payer (e.g., hospital visits, diagnostic tests, inpatient days), we used institutional tariffs and official price schedules from the University Hospital of Navarra and the regional public system (prices excluding taxes). For services provided by private institutions (e.g., hippotherapy, Therasuit, Method Peto), which are part of the direct expenses incurred by caregivers, we used the amounts reported by caregivers, corroborated with invoices, receipts, or price lists from providers when available. Private sector prices reflect the full comprehensive fees charged to families, including professional fees, materials, and use of facilities. Unit costs and unit definitions (e.g., per session, per diagnostic test, per inpatient day) are detailed in Supplementary Table S1, which specifies the measurement unit and data source for each item. Most unit costs were obtained from official tariffs of the University Hospital of Navarra, public price lists (BON), and validated out-of-pocket data from private centers, as described in our previous cost-of-illness study in pediatric cerebral palsy (10.3389/fpubh.2025.1589114) [17], which includes the full price lists and references. All costs are expressed in 2023 euros, updated to that year using the Spanish Consumer Price Index (CPI); where applicable in the economic evaluation, a 3% annual discount rate was applied to costs (and QALYs) in accordance with national guidelines [22].

#### Categorisation and prevention of double counting

Intervention costs (IERT) correspond to privately or semi-subsidized Intensive and Emerging Rehabilitation Therapies acquired outside the public healthcare system. These were valued as integral service prices, already including all components of the therapy (professional fees, materials, facilities, and medical supervision), without adding sub-costs. In contrast, Other Healthcare Costs represent the publicly funded Standard Treatment (ST) received by all participants—routine rehabilitation, hospitalizations, diagnostic tests, medical consultations, and medications. Both categories (IERT and public healthcare) were defined a priori in the EBQ-CP questionnaire and recorded in separate sections, ensuring that the same resource could not appear in both. Costs were computed as quantity × unit price, and a hierarchical reconciliation rule was applied to prevent double counting: administrative records were used for publicly funded items, and caregiver data for private or out-of-pocket components. For analytical purposes, unit prices for healthcare services and therapies were assumed to remain stable within the 12-month observation period, reflecting standard practice in short-term health economic evaluations where *intra-annual price variations are negligible*. Thus, all participants received the standard public healthcare package, while the IERT group additionally incurred private or institutionally subsidized therapy costs; total IERT costs therefore combine public healthcare expenditures with privately financed components, whereas Standard Treatment costs reflect only publicly funded care. This structure ensures that cost categories remain mutually exclusive and non-overlapping.

### Intervention and comparators

The primary comparison assessed the cost-effectiveness of standard treatment alone versus standard treatment supplemented with Intensive and Emerging Rehabilitation Therapies (IERT). Within the IERT arm, we examined two overlapping subgroups: Intensive Rehabilitation Therapies (IRT) and Emerging Rehabilitation Therapies (ERT). *These subgroups are not mutually exclusive;* some participants received therapies classified in both categories. This overlap is analytically relevant and is retained to reflect real-world clinical practice rather than impose artificial group separation. In addition, we conducted a secondary analysis stratified by individual therapy type. This was done to account for the substantial heterogeneity in intervention modality, frequency, and cost profiles across therapies, and to reduce aggregation bias that might otherwise obscure clinically meaningful differences.

Standard treatment consisted of conventional physical rehabilitation therapies, along with pharmacological and surgical care, provided within the public healthcare system. Intensive Rehabilitation Therapies (IRT) included intensified physical, speech, and occupational therapy programs. Emerging Rehabilitation Therapies (ERT) comprised motor and sensory learning interventions such as hippotherapy, the Petö method, and Therasuit, as well as complementary or non-conventional approaches (e.g., homeopathy).

The frequency and intensity of each intervention were based on individualized rehabilitation plans delivered at the University Hospital of Navarra and complementary services accessed privately. Participants were categorized into two groups according to the care they received: (1) a group receiving standard treatment plus one or more *Intensive and Emerging Rehabilitation Therapies* (*n* = 92), and (2) a group receiving standard treatment only (*n* = 56). Although the overall study period spanned 18 months (May 2023 to December 2024), the active economic and effectiveness analyses were restricted to a 12-month observation window corresponding to the effective period of treatment exposure for all participants. The 18-month duration covered the full operational timeline of the study, including recruitment, baseline data validation, and administrative follow-up, but only 12 months reflected actual treatment activity.

Specifically, the July–September school-holiday months were excluded from the cost and effectiveness analyses because therapy sessions were markedly reduced or paused during this period, both in public and private settings. In the public system, most standard therapies are delivered through school-based care programs coordinated with educational centers, which close during the summer. Consequently, these services are systematically suspended from July to September. In parallel, families commonly interrupt private therapy sessions during this time, reporting that summer serves as a rest period after continuous rehabilitation throughout the year. Additionally, many of the collaborating private or semi-private rehabilitation centers temporarily close or reduce their activity during this period. The exclusion of these non-active months was uniformly applied to all participants and aimed to avoid artificially lowering treatment intensity or diluting mean annual costs and outcomes. This methodological adjustment ensures consistency across groups and accurately reflects real-world service delivery patterns in pediatric rehabilitation in Spain. Such handling of inactive intervention periods is consistent with health economic methodological recommendations [30, 31].

### Effectiveness assessment and cost-effectiveness analysis

Effectiveness was assessed using the EQ-5D-Y proxy version, completed by caregivers. This preference-based instrument captures HRQoL across five dimensions (mobility, self-care, usual activities, pain/discomfort, and anxiety/depression), each with three severity levels. Utility values were derived from the Spanish EQ-5D-Y value set [25, 32]. QALYs were estimated using the area under the curve method, based on EQ-5D-Y scores collected at baseline, 6 months, and 12 months [19]. Linear interpolation was assumed between measurement points. Building on the 12-month real-world data, an individual-level long-term microsimulation model was developed to project lifetime costs and QALYs for each patient. This modelling framework extrapolates beyond the observed period, accounts for patient-level heterogeneity in disease trajectories, and enables a comprehensive estimation of long-term cost-effectiveness. In addition to EQ-5D-Y utilities, the Gross Motor Function Classification System (GMFCS) was used to stratify patients by severity of motor impairment. This five-level system, with higher levels indicating more severe disability, provides relevant clinical information associated with differences in HRQoL, healthcare utilization, and cost. In this study, GMFCS levels were used specifically within the long-term microsimulation model to inform health state transitions and associated parameters [33].

Cost-effectiveness was evaluated by including participants with complete cost and effectiveness data. Incremental cost-effectiveness ratios (ICERs) were calculated by dividing the mean difference in total costs between the group combining *Intensive and Emerging Rehabilitation Therapies* with standard treatment and the standard treatment-only group by the corresponding difference in QALYs.

### Statistical Analysis

#### Short-term analysis: seemingly unrelated regression (SURE)

To estimate the short-term cost-effectiveness of *Intensive and Emerging Rehabilitation Therapies*, we used a regression-based approach based on observational data collected over a 12-month period. A system of equations was independently estimated for each specific therapy classified under the Intensive Rehabilitation Therapies (IRT) and Emerging Rehabilitation Therapies (ERT) categories, each considered as an addition to standard treatment. These models estimated total healthcare costs, combining the costs associated with standard care (HC) and those associated with each type of non-standard therapy.

Importantly, the SURE framework treats each therapy as a distinct analytical unit, allowing us to estimate costs and outcomes for each therapy individually. Consequently, in the short-term analysis, we report cost-effectiveness (ICERs) for each specific therapy plus Standard Treatment versus Standard Treatment alone. We do not report a pooled ICER for groups or subgroups (IERT, IRT, ERT) because aggregating heterogeneous and partially overlapping interventions could introduce bias and produce misleading results. Subgroup results (ERT and IRT) are presented for exploratory purposes only and are not additive, as some patients receive therapies in both subgroups; within each subgroup, each patient is considered only once. The overlap between subgroups is detailed in Supplementary Table S1. The equations were specified as follows: *Intensive Rehabilitation Therapies (IRT)*

$$\begin{aligned} \:{Costs\:(HC+IRT)}_{CTi} &=\:{\alpha\:}_{0}+{SpeechTH\:\alpha\:}_{1}+{Rehab\:\alpha\:}_{2}+{OccupTH\:\alpha\:}_{3}+{u}_{1i}\\ {Effect}_{CTi} &=\:{\beta\:}_{0}+{SpeechTH\:\beta\:}_{1}+{Rehab\:\beta\:}_{2}+{OccupTH\:\beta\:}_{3}+{u}_{2i} \end{aligned}$$

*Emerging Rehabilitation Therapies (ERT)*

$$\begin{aligned} {Costs\:(HC+ET)}_{ATi}&=\:{\alpha\:}_{0}+{HippoTH\:\alpha\:}_{1}+{Home\:\alpha\:}_{2}+{PetMth\:\alpha\:}_{3}+{Ther\:\alpha\:}_{4}+{u}_{1i}\\ {Effect}_{ATi} &=\:{\beta\:}_{0}+{HippoTH\:\beta\:}_{1}+{Home\:\beta\:}_{2}+{PetMth\:\beta\:}_{3}+{Ther\:\beta\:}_{4}+{u}_{2i} \end{aligned}$$

Where *SpeechTH*,* Rehab*,* OccupTH*,* HippoTH*,* Home*,* PetMth*, and *Ther* are binary indicators equal to 1 if the child receives the corresponding therapy (Speech therapy, Rehabilitation, occupational therapy, hippotherapy, homeopathy, Petö method, Therasuit, respectively), in addition to standard treatment, and 0 otherwise. *Effect i* represents the QALYs gained per individual *i.* The systems were estimated using the Seemingly Unrelated Regression Estimator (SURE), which accounts for correlations between cost and effect equations arising from shared unobserved factors [34]. This approach enables joint estimation of ICERs and their uncertainty, represented via cost-effectiveness acceptability curves (CEACs) and confidence ellipses at 50%, 75%, and 95% levels [35].

#### Long-term analysis: individual-level microsimulation model

##### Model structure

To complement the short-term regression-based analysis and project long-term outcomes, we developed a first-order individual-level microsimulation model in R [36]. The model simulates a cohort of 100,000 hypothetical individuals with CP over a 30-year horizon using annual cycles. An individual microsimulation structure (rather than a Markov cohort model) was chosen because of the need to capture the inter-individual heterogeneity and variable clinical trajectories unique to the paediatric CP population [37]. This approach allows for a more realistic representation of the clinical course, as well as stratified GMFCS simulations [36, 38].

##### Health states and transitions

Individuals transition through four mutually exclusive health states defined by the Gross Motor Function Classification System (GMFCS): GMFCS I–II (walks without limitations), GMFCS III (walks with limitations or uses assistive devices), GMFCS IV–V (non-ambulatory or uses a wheelchair), and Dead. At baseline, individuals enter the model in the health state corresponding to their observed GMFCS level (I–V), reflecting the severity distribution of the study cohort. Health state transitions are governed by fixed annual probabilities derived from severity-specific mortality and disease progression rates and are simulated stochastically using Monte Carlo sampling [39].

##### Treatment strategies and cycle-based parameters

Per-cycle costs and utility weights were assigned according to health state and treatment strategy. The model incorporated four mutually exclusive strategies — that is, each simulated individual was assigned to only one treatment pathway during a cycle: no treatment, Standard Treatment (ST), ST plus Intensive Rehabilitation Therapies (ST + IRT), and ST plus Emerging Rehabilitation Therapies (ST + ERT). To ensure methodological robustness and consistency with the short-term analysis, two complementary modelling scenarios were implemented. *The first aggregated therapies into the four strategies described above* to test the sensitivity of long-term health state transitions and utilities — effects that are often more evident over extended horizons. Grouping interventions at this level follows standard microsimulation practice in heterogeneous chronic populations [36, 39], allowing evaluation of how disease severity progression and treatment type jointly influence long-term cost-effectiveness, while also supporting policy-relevant interpretation and system-level decision-making. *The second scenario disaggregated results by each individual therapy*, using an analytical structure consistent with the short-term analysis, to ensure methodological coherence and enable identification of the most cost-effective interventions within each category. This granular approach is particularly relevant in CP, where overlapping therapeutic use and patient heterogeneity may influence cost-effectiveness outcomes. Together, these complementary approaches balance robustness and interpretability across time horizons, ensuring internal consistency and reliability of long-term projections.

##### Technical implementation and outputs

Model inputs were derived from two sources: real-world data from our observational cohort (used for costs, utility values, and treatment distributions) and published literature (used for mortality, progression, and transition probabilities [1, 37, 40]). All parameter values and sources are provided in Table S2 of the supplementary material. The model follows a modular structure to ensure transparency and reproducibility. The core functions include: *Probs()* to estimate transition probabilities, *Costs()* to accumulate direct medical costs, *Effs()* to calculate QALYs, and *MicroSim()* to simulate individual-level disease trajectories under each strategy. A common random seed is set across strategies to ensure that each simulated individual retains identical baseline characteristics, thus isolating the effect of treatment from stochastic variation. Key outputs include discounted costs and QALYs, health state transition matrices, and individual-level traces over all cycles [38, 39]. This modelling approach supports stable and consistent estimation of long-term cost-effectiveness outcomes and allows subgroup analysis by GMFCS severity.

**Fig. 2:**
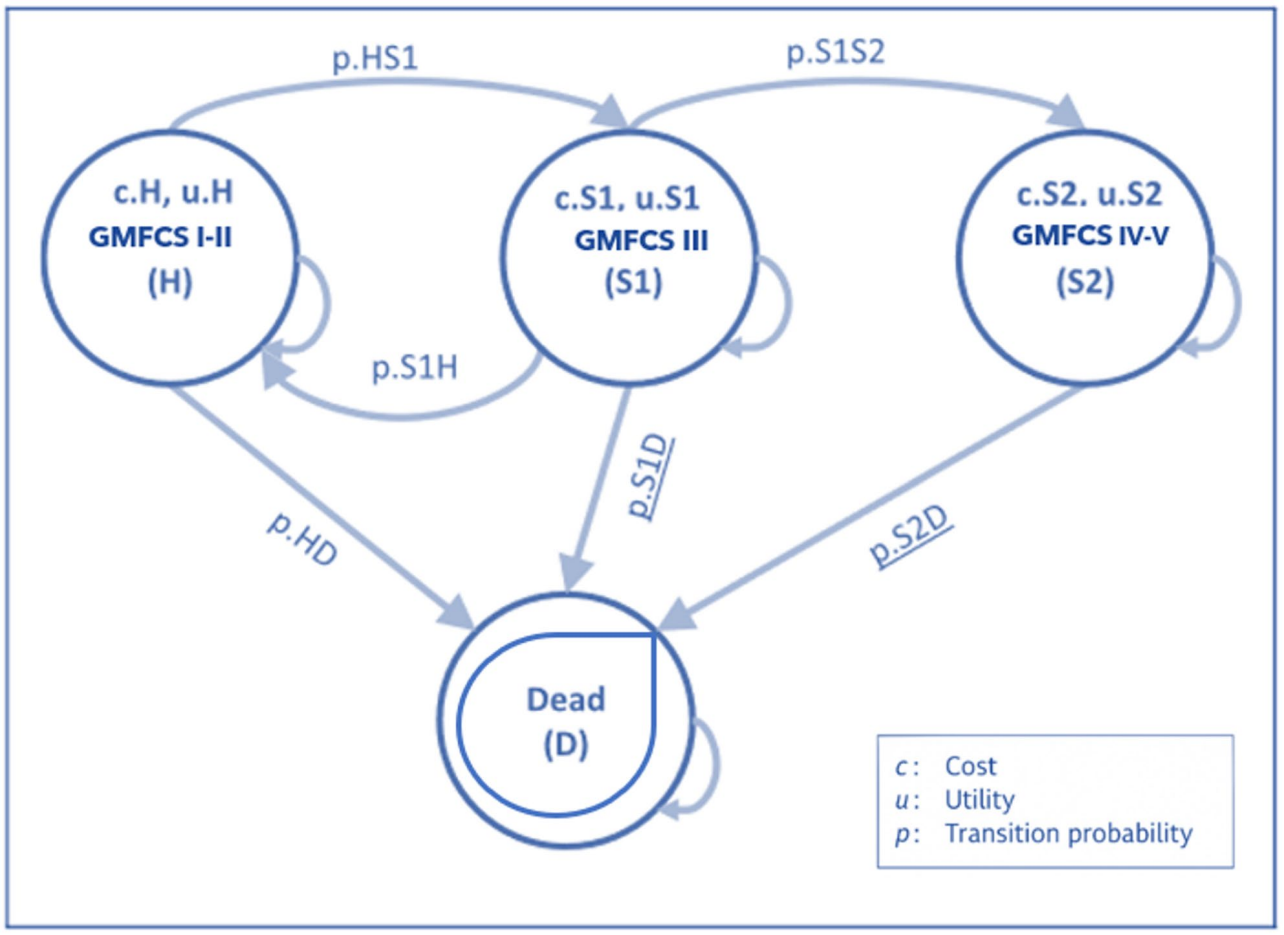
Schematic representation of the GMFCS-based mobility health-state microsimulation model. Health statuses: GMFCS I-II (walks without limitations: H), GMFCS III (walks with limitations or uses assistive devices: S1), GMFCS IV-V (non-ambulatory or uses a wheelchair: S2), Dead (D)

This hybrid modelling approach, which combines short-term real-world data with long-term microsimulation, provides a comprehensive assessment of the cost-effectiveness of Intensive and Emerging Rehabilitation Therapies in pediatric CP. Full technical documentation and R code are available in the supplementary material and GitHub repository (see Fig. 2, Appendix A, and Table S2). Figure 2 illustrates a conceptual framework adapted from prior studies [36, 38, 39], modified to align with the specific objectives of this research and constructed using our own observational data.

#### Missing data and completeness

EQ-5D-Y proxy assessments at baseline, 6, and 12 months were fully observed (0% missing). Cost diaries and covariates showed very high completion; denominators are reported in the tables. Overall attrition was 5/148 (3.4%) and was confined to the ERT subgroup within the IERT arm (5/92); no losses occurred in the IRT subgroup or in the standard-care arm. Given the near-complete outcomes and the small, subgroup-limited attrition, the primary analysis followed a complete-case approach including participants with complete cost and outcome data. Multiple imputation was not performed because it would add little value under these conditions.

### Sensitivity analysis

To assess uncertainty in the cost-effectiveness estimates, two complementary probabilistic sensitivity analyses were performed, corresponding to the short- and long-term components of the study. *In the short-term (12-month) analysis*, a model-based PSA was implemented using the *Seemingly Unrelated Regression Equations (SURE)* framework. This method jointly estimates the cost and effect equations while accounting for their covariance structure, allowing for empirical simulation of uncertainty without assuming parametric error distributions. The resulting variance-covariance matrices were used to derive confidence ellipses and cost-effectiveness acceptability curves (CEACs). *In the long-term (30-year) microsimulation model*, a fully probabilistic sensitivity analysis was performed using non-parametric bootstrap resampling (1,000 Monte Carlo iterations). Parameter uncertainty in transition probabilities, utilities, and costs was propagated through the model. This analysis generated the cost-effectiveness plane (Fig. 4), illustrating the distribution of incremental cost-utility pairs over time.

All analyses were implemented in R (v4.3.2), with complementary data processing in Excel and Gretl (v1.9.4).

### Ethical considerations

The EPCINA study received approval from the Navarra Research Ethics Committee (CEIm) (PI_2023/46) and was registered with the Navarra Clinical Research Secretariat. Written informed consent was obtained from parents and children over 12 years of age able to understand the purpose of the study.

## Results

**Fig. 3:**
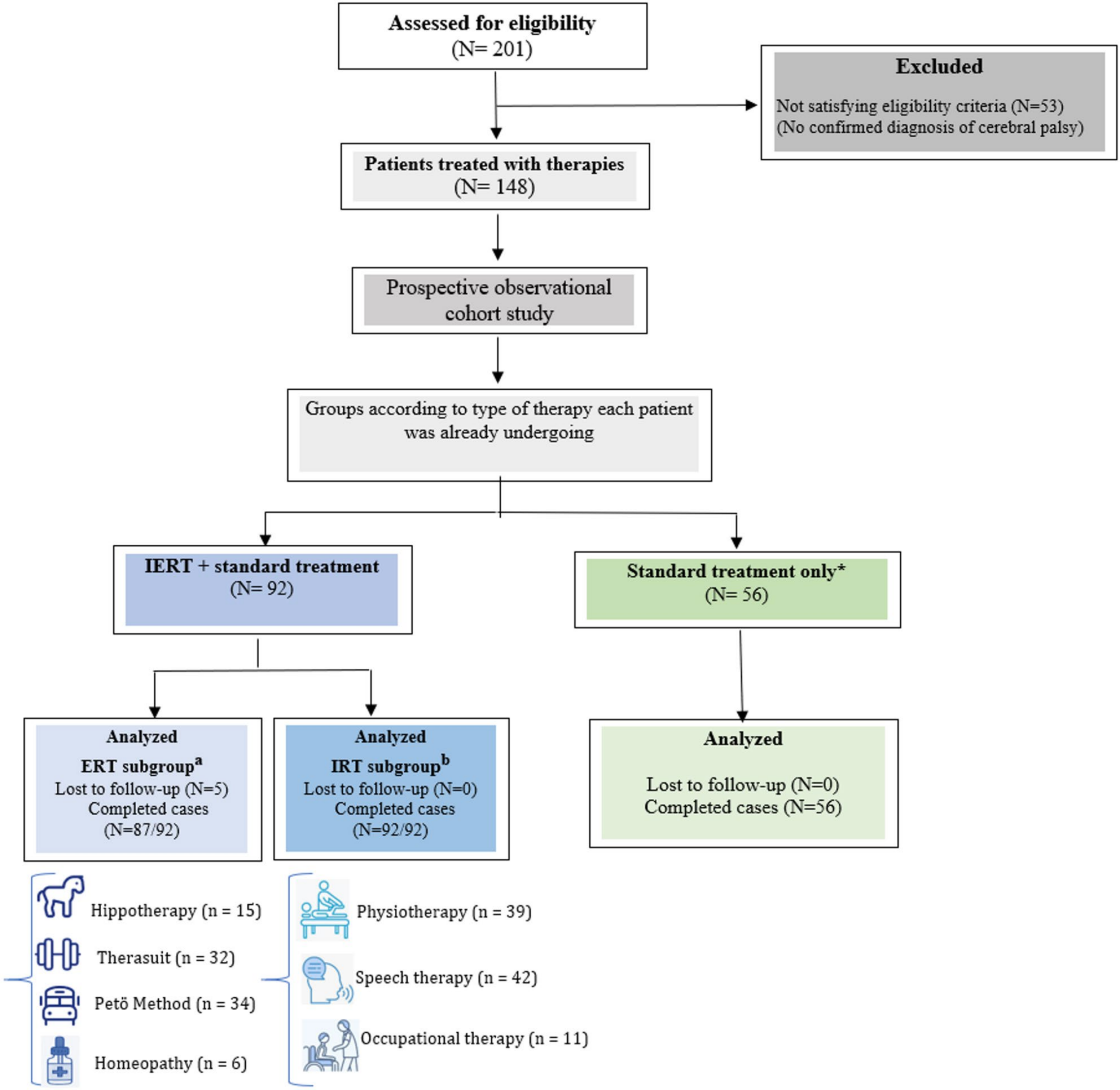
Flowchart of Patient Selection and Follow-up in the Cost-Effectiveness Study. IERT: Intensive and Emerging Rehabilitation Therapies. ᵃ ERT subgroup: Subgroup of the IERT group receiving Emerging Rehabilitation Therapies in addition to standard treatment. Includes Hippotherapy, Therasuit, the Petö method, and Homeopathy. Note: 5 children in this subgroup *discontinued ERT therapy*. ᵇ IRT subgroup: Subgroup of the IERT group receiving Intensive Rehabilitation Therapies in addition to standard treatment. Includes physiotherapy, speech therapy, and occupational therapy. * Standard treatment: Refers to standard clinical practice in the treatment of children with CP, includes conventional rehabilitation approaches aimed at maintaining or improving motor function, communication, and daily activities

We analysed data from 148 children with cerebral palsy (78 boys and 70 girls), aged between 3 and 18 years (mean age: 9.72 ± 4.22 years). Distribution across GMFCS levels was: I (*n* = 24), II (*n* = 35), III (*n* = 29), IV (*n* = 21), V (*n* = 39), ensuring a representative spread of severity. As illustrated in Figs. 3 and 92 children received Intensive and Emerging Rehabilitation Therapies plus standard care and 56 received standard care alone. EQ-5D-Y was completed at baseline, 6, and 12 months (0% missing). Overall attrition was 5/148 (3.4%), confined to the ERT subgroup within the IERT arm (5/92); no losses occurred in the IRT subgroup or in the standard-care arm (see flow diagram). This well-distributed and near-complete dataset enabled robust therapy-specific cost-effectiveness estimation across severity levels and strategies.

Table 1 summarizes the sociodemographic and clinical characteristics of participants, disaggregated by treatment group. The dataset was complete across all relevant variables, including age, sex, household income, socioeconomic index by geographic area, type of cerebral palsy, and GMFCS level. Overall, 53% of participants were male, 42% belonged to households earning between €31,000 and €51,000 annually, and 45% resided in areas with a medium socioeconomic index. Spastic cerebral palsy accounted for the majority of cases (79%).

**Table 1:** Baseline Characteristics of Children and Their Families by Therapy Group (*n* = 148)

Characteristic	Total	IERT + Standard Care	Standard Care
	(*N* = 148), n (%)	(*N* = 92), n (%)	(*N* = 56), n (%)
Age (years)			
3 to 6	45 (31%)	24 (26%)	21 (38%)
7 to 12	60 (41%)	39 (42%)	21 (38%)
13 to 18	43 (29%)	29 (32%)	14 (24%)
Family income			
Less or up €30,000	28 (19%)	15 (16%)	13 (23%)
€31,000 to €52,000	61 (42%)	40 (43%)	21 (38%)
€53,000 to €72,000	49 (32%)	30 (33%)	19 (34%)
More than €73,000	10 (7%)	7 (8%)	3 (5%)
Socioeconomic index for area			
Low	23 (16%)	11 (12%)	12 (21%)
Low-Medium	41 (28%)	25 (27%)	16 (29%)
Medium	68 (45%)	48 (52%)	20 (36%)
High	16 (11%)	8 (9%)	8 (14%)
Sex			
Female	70 (47%)	46 (50%)	24 (43%)
Male	78 (53%)	46 (50%)	32 (57%)
Type of Cerebral Palsy			
Spastic	117 (79%)	67 (73%)	50 (89%)
Non-spastic (ataxic, dyskinetic and mixed)	31 (21%)	25 (27%)	6 (11%)
GMFCS Level			
Level I	24 (16%)	7 (8%)	17 (30%)
Level II	35 (24%)	16 (17%)	19 (34%)
Level III	29 (20%)	20 (22%)	9 (16%)
Level IV	21 (14%)	18 (20%)	3 (6%)
Level V	39 (26%)	31 (34%)	8 (14%)

*IERT* Intensive and Emerging Rehabilitation Therapies. This group contains two subgroups: ERT, patients taking Emerging Rehabilitation Therapies and IRT, patients taking Intensive Rehabilitation Therapies. Data are n (%), calculated over non-missing observations. Denominators for each column are shown in the headers. No missing values were observed for variables listed in this table

Children receiving Intensive and Emerging Rehabilitation Therapies in addition to standard care were more frequently classified within GMFCS levels III to V (75%), whereas those in the standard care group were predominantly in levels I and II (64%). This distribution likely reflects greater therapy needs among children with higher motor severity. Recognizing this, we stratified analyses by GMFCS level where appropriate to account for variations in baseline utility and healthcare utilization. All severity levels were sufficiently represented to enable meaningful comparisons within the cost-effectiveness framework.

### Costs

Healthcare costs were higher when Intensive and Emerging Rehabilitation Therapies (IERT) were added to standard treatment. Table 2 presents mean annual healthcare costs per child, distinguishing between intervention costs (IERT + ST) and those funded by the public healthcare system (Standard Treatment). Supplementary Table S1 provides detailed unit costs, quantities, and sources used to estimate these averages.

**Table 2:** Annual treatment costs (€2023), public healthcare system perspective. Main comparison: Standard Treatment versus IERT + Standard Treatment

Resource Category	Mean annual cost (€)	
	IERT + Standard Treatment (€) Intervention ᵃ (*n* = 92)	Standard Treatment (€) Comparator ᵇ (*n* = 56)
Intensive and Emerging Rehabilitation Therapies (IERT)	**5**,**683**	**0**
Healthcare Costs (from Public Healthcare system)	**5**,**396**	**5**,**156**
General practitioner care	46	47
Specialist medical care	202	196
Diagnostic Tests	219	222
Hospitalisation	735	643
Medication	1082	878
Standard Therapies	3352	3170
Total Healthcare Costs	**11**,**079**	**5**,**156**
Base-case incremental Cost (IERT + ST vs. ST) **ᶜ**	*5923*	

Data are mean annual values (€2023). n denotes non-missing observations; column denominators are shown in the headers

^a^IERT = Intensive and Emerging Rehabilitation Therapies, comprising two partially overlapping subgroups: (1) ERT = Emerging Rehabilitation Therapies (hippotherapy, Petö Method, Therasuit, homeopathy); and (2) IRT = Intensive Rehabilitation Therapies (physiotherapy, speech therapy, occupational therapy). Overlap refers to patients who receive therapies from both subgroups; there is no duplication of costs, since a patient may undergo more than one therapy between subgroups, but not within the same subgroup, each patient is counted only once in each subgroup

^b^ST = Standard Treatment

^c^Base-case ΔCost (IERT + ST − ST) = difference in total annual healthcare costs between the intervention and comparator groups (e.g., 11,079 − 5,156 = 5,923€). Intervention (IERT) and public healthcare costs were defined as mutually exclusive categories and valued using the hierarchical reconciliation rule described in Sect. 2.3.2. Detailed unit costs, quantities, and subgroup breakdowns are provided in Supplementary Table S1-2b

* Five participants in the ERT subgroup were lost to follow-up; the ERT cost analysis used *n* = 87 complete cases (see flow diagram)

Bold values are used to highlight key cost outcomes, including total costs and the base-case incremental cost, to improve readability and facilitate interpretation

The mean annual cost per child increased from €5,156 for standard treatment alone to €11,079 for those receiving IERT + ST, resulting in an incremental annual cost of €5,923 (Table 2). These averages represent real-world expenditures per patient rather than cumulative group totals, thus avoiding double counting between overlapping subgroups. Within the IERT group, the mean annual intervention-related cost was €5,683 per child, corresponding to therapy-specific expenses reported as total service prices in private or semi-subsidized centers. Public healthcare costs (Standard Treatment) for this group averaged €5,396, including medical visits, hospitalizations, diagnostic tests, medications, and standard rehabilitation sessions.

Among individual healthcare components, medication represented the largest cost category (€1,082 per child annually), followed by hospitalizations (€735), specialist consultations (€202), and diagnostic tests (€219). Standard rehabilitation therapies were slightly more costly in the IERT group (€3,352 vs. €3,170), likely reflecting higher therapy intensity in children with more complex needs.

Supplementary Table S1 presents the complete breakdown of unit costs and clarifies that, within the IERT + ST group (*n* = 92), the same patient could receive therapies belonging to both subgroups (ERT and/or IRT), but no more than one within the same subgroup. Thus, each patient was counted only once per subgroup, ensuring that there was no duplication of costs either within or between subgroups.

### Cost-effectiveness analysis

#### Short-term horizon

Therapies varied considerably in their cost-effectiveness profiles. ICERs were calculated for each specific therapy plus Standard Treatment versus Standard Treatment alone, following the pre-specified SURE approach to avoid aggregation bias; no pooled ICERs for intervention groups (IERT) or subgroups (IRT, ERT) are reported. Therasuit and physiotherapy emerged as the most efficient interventions, achieving meaningful gains in QALYs at moderate incremental costs (Table 3). Therasuit therapy resulted in a mean gain of 0.222 QALYs, with an associated incremental cost-effectiveness ratio (ICER) of €18,830 per QALY. Physiotherapy produced a slightly lower QALY gain (0.216) but remained within the accepted cost-effectiveness range, with an ICER of €31,772 per QALY. The effectiveness of physiotherapy was statistically significant at the 95% level (*p* < 0.001), while Therasuit showed marginal significance at the 90% level (*p* = 0.0756).

In contrast, several therapies yielded unfavourable cost-effectiveness profiles. Occupational therapy, speech therapy, and hippotherapy incurred higher costs while producing no measurable improvement or even a decline in QALYs, compared to standard treatment. These interventions were classified as dominated, indicating that they are more expensive and less effective than the comparator. The Petö Method and homeopathy produced modest QALY gains (0.066 and 0.058, respectively), but their ICERs exceeded €65,000 per QALY, and neither effect was statistically significantly different from zero with respect to effectiveness.

These findings suggest that, under commonly accepted willingness-to-pay thresholds in Spain (€25,000–€60,000 per QALY) [21, 23], only Therasuit and physiotherapy represent economically attractive additions to standard care for children with cerebral palsy. The wide variation in both cost and clinical benefit across therapies highlights the importance of evidence-based prioritization when integrating Intensive and Emerging Rehabilitation Therapies into pediatric neurorehabilitation strategies and public funding decisions.

**Table 3:** Short-term cost-effectiveness (12-month SURE analysis): Intensive and Emerging Rehabilitation Therapies vs. standard treatment (Spanish public payer; 2023 €)

Therapy	Cost (€) Mean (SD)	*P*-value	Effect (QALY) Mean (SD)	*P*-value	ICER (€/QALY)
Standard treatment (*n* = 56)	5,156.48 (354.22)	< 0.001	0.517 (0.03)	< 0.001	-
*Intensive Rehabilitation therapies* (*n* = 92)					
Occupational therapy (*n* = 11)	9,258.25 (874.22)	< 0.001	−0.067 (0.08)	0.4539	Dominated
Physiotherapy (*n* = 39)	6,863.01 (552.85)	< 0.001	0.216 (0.05)	< 0.001	€ 31,772/QALY
Speech therapy (*n* = 42)	7,997.73 (541.08)	< 0.001	−0.081 (0.05)	0.1435	Dominated
*Emerging Rehabilitation therapies* (*n* = 87)					
Hippotherapy (*n* = 15)	5,130.24 (703.30)	< 0.001	−0.046 (0.08)	0.5806	Dominated
Therasuit (*n* = 32)	4,193.84 (1,044.10)	< 0.001	0.222 (0.12)	0.0756	€ 18,830/QALY
Petö method (*n* = 34)	4,338.16 (522.27)	< 0.001	0.066 (0.06)	0.287	€ 65,226/QALY
Homeopathy* (*n* = 6)	4,898.51 (532.65)	< 0.001	0.058 (0.06)	0.3554	€ 83,238/QALY

Pre-specified short-term approach: therapy-specific ICERs versus Standard to avoid aggregation bias; subgroup (ERT/IRT) summaries are exploratory and non-additive

ICER = incremental cost-effectiveness ratio. Estimates from 12-month SURE. “Dominated” indicates more costly and less effective than Standard. Analyses are complete-case; *n* denotes participants with non-missing costs and EQ-5D-Y. Attrition occurred only in the ERT subgroup (5/92) → ERT *n* = 87; no losses in IRT or Standard (see Fig. 3, flow diagram). No multiple imputation was undertaken due to near-complete data and subgroup-limited attrition

*Some therapy-specific subgroups have small sample sizes (e.g. *n* = 6 for homeopathy); therefore, statistical significance—particularly for cost outcomes—should be interpreted with caution despite low *p*-values. Observed significance reflects consistent cost differences across therapies combined with relatively low within-group cost variability

#### Long-term horizon: individual-level microsimulation model

To assess the long-term cost-effectiveness of each strategy, an individual-level microsimulation model projected lifetime healthcare costs and QALYs over a 30-year horizon. Compared to a no-treatment scenario, all active strategies improved health outcomes at higher costs, placing them in the northeast quadrant of the cost-effectiveness plane (Table 4).

*Standard treatment* yielded 1.464 additional QALYs at an incremental cost of €50,173, with an ICER of €34,280 per QALY. *Intensive Rehabilitation Therapies* aggregating speech therapy, physiotherapy, and occupational therapy provided the greatest QALY gains (2.437), though at a higher cost (€124,750), resulting in an ICER of €51,197. *Emerging therapies* (Therasuit, Petö, Homeotherapy, and Hippotherapy) produced 1.950 QALYs at an incremental cost of €95,509, leading to an ICER of €48,975. To understand within-group variation, each therapy was independently evaluated against standard treatment. Among *Intensive Rehabilitation Therapies*,* speech therapy* was the most efficient, generating 6.83 QALYs at an ICER of €20,451, followed by *physiotherapy* (4.92 QALYs; ICER €25,789) and *occupational therapy* (4.69 QALYs; ICER €30,199). All remained within acceptable cost-effectiveness thresholds, supporting their continued integration into standard care.

Among emerging therapies, Therasuit achieved the greatest health benefit, gaining 5.49 QALYs with an incremental cost of €70,889, resulting in the most favourable ICER (€12,922). The Petö method generated 4.31 additional QALYs (ICER: €14,774), followed by homeopathy (4.22 QALYs; ICER: €18,160) and hippotherapy (3.67 QALYs; ICER: €13,784). Although hippotherapy presented a lower ICER compared to some therapies, its health benefits were comparatively smaller. Overall, all Emerging Rehabilitation Therapies demonstrated acceptable cost-effectiveness profiles relative to standard treatment.

**Table 4:** Cost-effectiveness results from the individual-level microsimulation model over 30 years from the perspective of the Spanish public healthcare system (€2023). Simulated cohort: 100,000 individuals

Comparison	Treatment	Costs (€)	MCSE Costs	QALYs	MCSE QALYs	Δ Costs (€)	Δ QALYs	ICER (€/QALY)
*(1) Grouped Strategies*	No Treatment	94,049	189	14.454	0.010	–	–	–
	Standard treatment	144,222	269	15.918	0.009	50,173	1.464	34,280
	Intensive rehab. Therapies	218,799	404	16.891	0.009	124,750	2.437	51,197
	Emerging rehab. Therapies	189,558	350	16.404	0.009	95,509	1.950	48,975
(2) Intensive Rehabilitation therapies vs. Standard	Occupational therapy	243,448	143	14.467	0.011	141,496	4.685	30,199
	Physiotherapy	228,784	140	14.700	0.011	126,832	4.918	25,789
	Speech therapy	201,010	152	14.626	0.012	99,058	6.833	20,451
*(3) Emerging Rehabilitation therapies vs. Standard*	Hippotherapy	153,399	139	9.758	0.011	50,510	3.664	13,784
	Therasuit	173,778	110	15.760	0.012	70,889	5.486	12,922
	Petö	166,610	101	14.588	0.011	63,722	4.313	14,774
	Homeopathy	179,437	109	14.490	0.011	76,548	4.215	18,160

Microsimulation at the individual level of a cohort of 100,000 people over a 30-year horizon, with an annual discount rate of 3% (costs and QALYs), from the perspective of the Spanish public payer (€2023). The analysis includes: (1) grouped strategies (no treatment, standard treatment, intensive rehabilitation therapies [IRT], and emerging rehabilitation therapies [ERT]); and (2) specific comparisons of therapies within IRT and ERT, both to capture patient-level heterogeneity, based on different parameters

*MCSE *Monte Carlo standard error, *ICER* Incremental cost-effectiveness ratio, *QALY *Quality-adjusted life years

### Sensitivity and uncertainty analysis

#### Long-term probabilistic analysis (30-year microsimulation model)

**Fig. 4:**
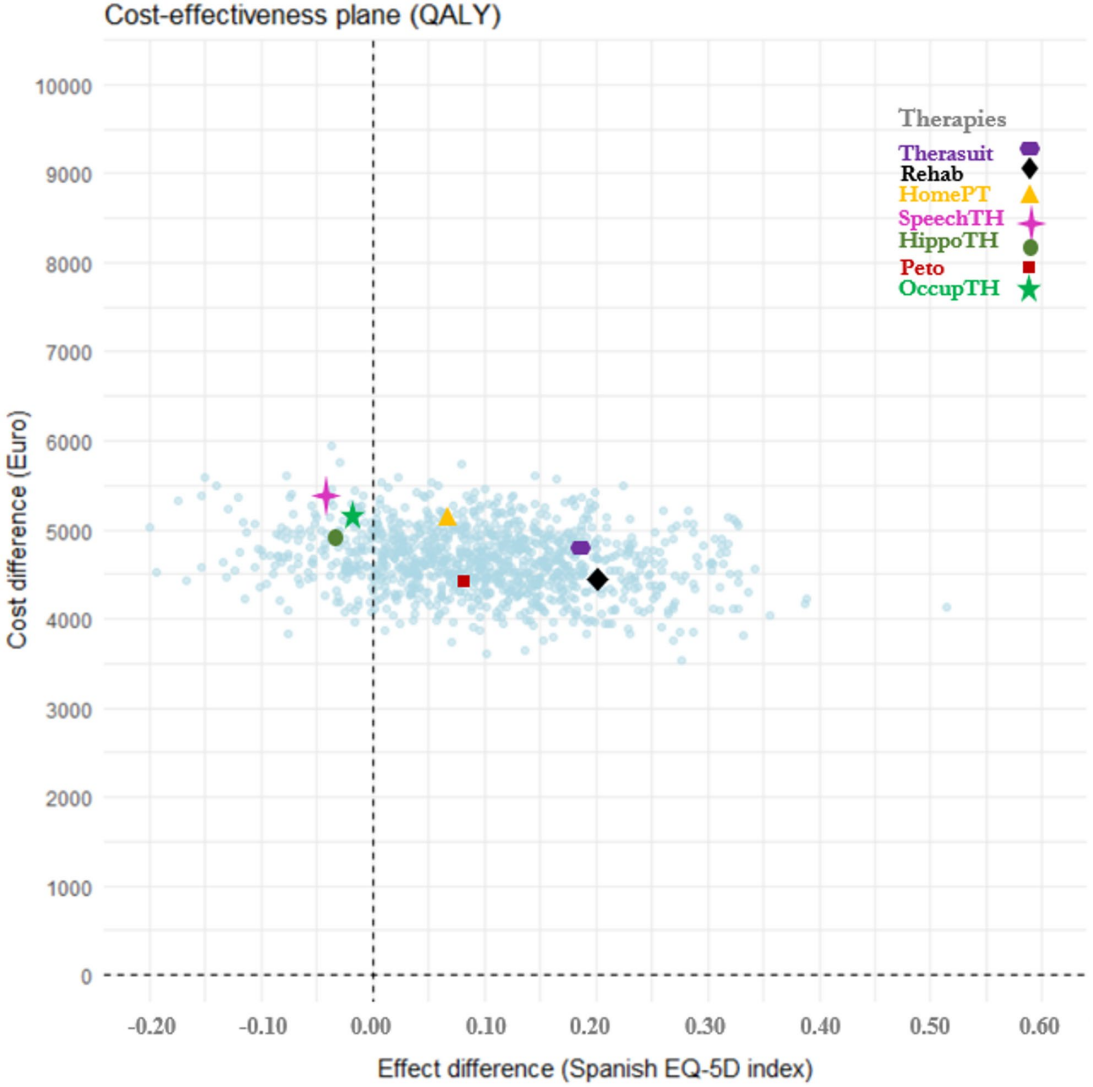
Cost-effectiveness plane of standard treatment vs. intensive and emerging rehabilitation therapies. (Long-term probabilistic sensitivity analysis, 30-year microsimulation model)

Figure 4 presents the cost-effectiveness plane derived from the bootstrap-based probabilistic sensitivity analysis. Each point represents a simulated pair of incremental costs and QALYs comparing IERT with standard treatment. Most simulations lie within the northeast quadrant, confirming that these therapies are generally more effective but also more costly than standard care. The narrow vertical dispersion indicates stable incremental costs, while the wider horizontal spread reflects variability in health gains and heterogeneity in treatment response.

*In the dynamic R-based model (not shown)*, therapies initially appearing near or left of the cost-effectiveness threshold gradually shifted rightward *after approximately five years*,* as cumulative health benefits increased.* This temporal shift suggests that interventions with moderate short-term effects may become cost-effective in the long term as sustained functional improvements accumulate.

#### Short-term probabilistic analysis (12-month data)

**Fig. 5:**
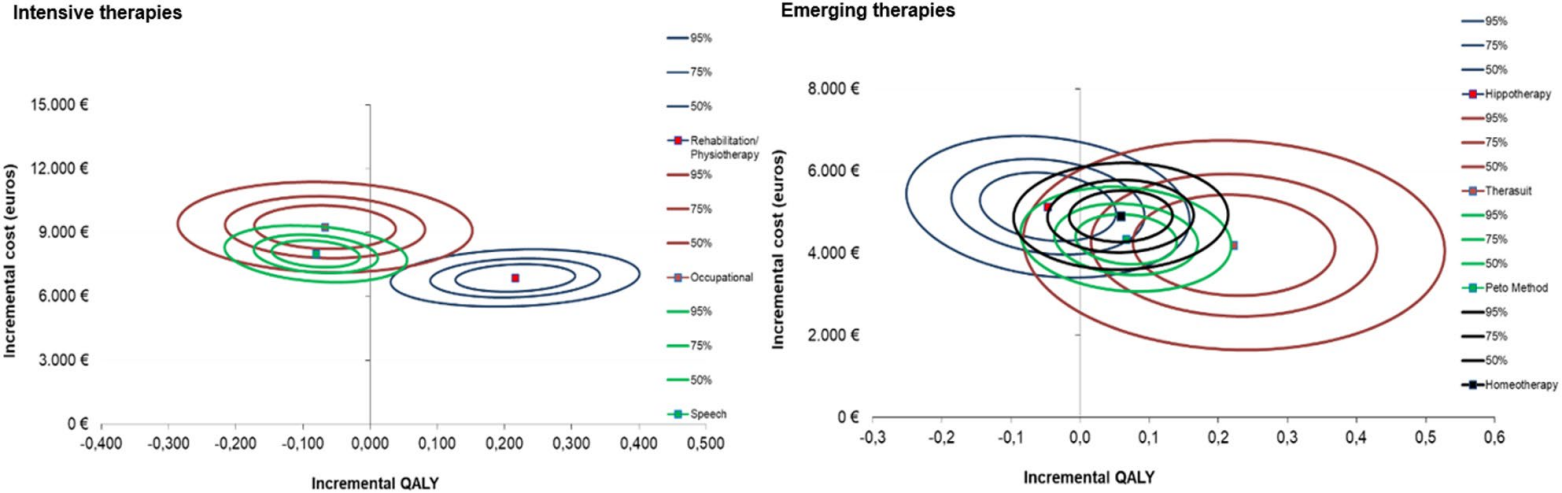
Confidence ellipses for intensive and emerging rehabilitation therapies (Short-term SURE-based probabilistic analysis, 12-month data)

**Fig. 6:**
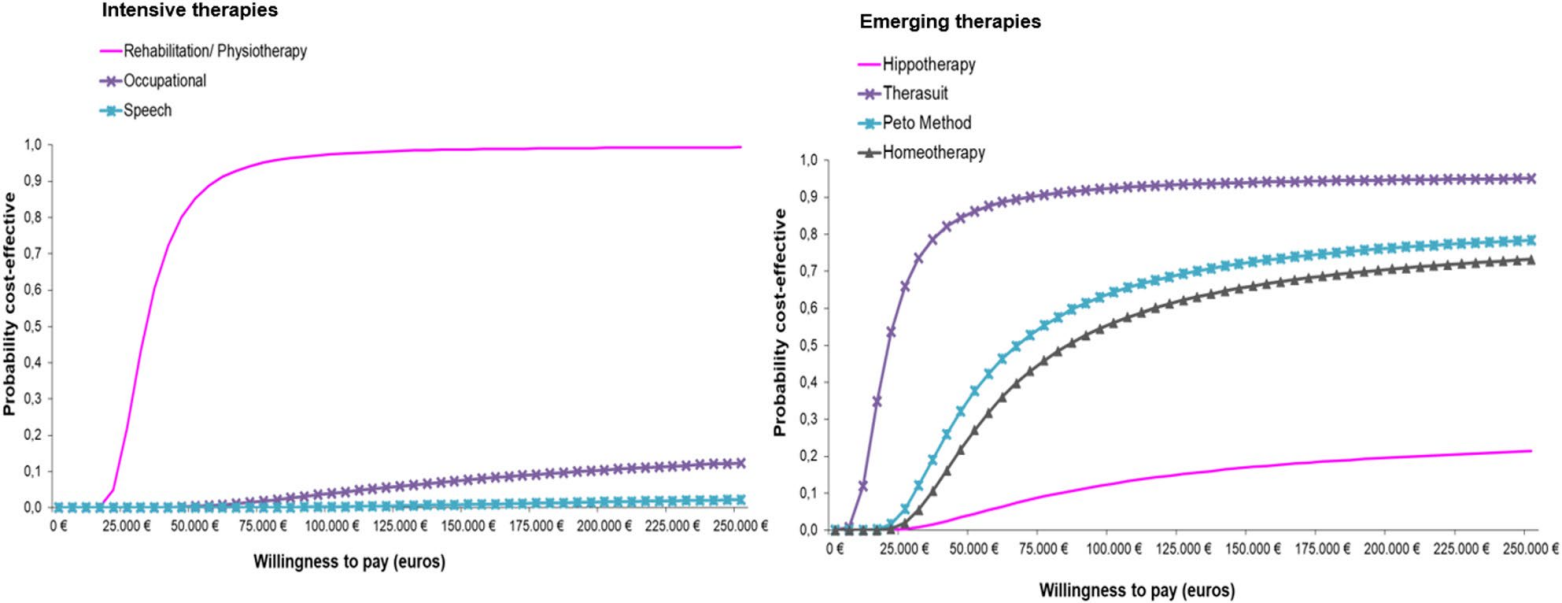
Cost-effectiveness acceptability curves of standard treatment versus intensive and emerging rehabilitation therapies (Short-term SURE-based probabilistic analysis, 12-month data)

Figure 5 shows the 50%, 75%, and 95% confidence ellipses for each therapy, reflecting the joint distribution of incremental costs and effects. Most therapies remained within the northeast quadrant, though the ellipse sizes varied, indicating different levels of precision and consistency across interventions. Figure 6 displays the corresponding cost-effectiveness acceptability curves (CEACs) for each therapy. Physiotherapy and Therasuit demonstrated the highest probabilities of cost-effectiveness, exceeding 85% at a €50,000 threshold. In contrast, the remaining therapies showed lower probabilities, with none surpassing 40% at this same threshold.

## Discussion

This study evaluated the cost-effectiveness of integrating *Intensive and Emerging Rehabilitation Therapies* with standard treatment in children with CP, *using real-world data* from a regional *observational cohort* combined with an *individual-level microsimulation model* to project *long-term outcomes*.

*In the short-term analysis*, based on real-world observational data and the SURE econometric model, Therasuit therapy and intensive physiotherapy had the most favourable cost-utility profiles. At a WTP threshold of €60,000 per QALY commonly used in Spain these therapies demonstrated high probabilities of being cost-effective—approximately 92% for physiotherapy and 87% for Therasuit. Other interventions, such as homeopathy and the Petö Method, showed smaller health improvements, whereas hippotherapy, occupational therapy, and speech therapy were less effective and more costly than standard treatment, thus representing dominated options in the short-term context. This heterogeneity highlights that not all IERT modalities contribute equally to quality-of-life improvement. In pediatric CP, where healthcare resources are constrained and out-of-pocket spending is substantial [14], identifying interventions with limited cost-effectiveness is essential to avoid inefficient allocation and inclusion of low-value treatments in care protocols [41, 42]. Moreover, because many of these therapies are partially financed by families, short-term cost-effectiveness differences also raise concerns about equity and access. Differences in affordability and coverage across socioeconomic groups should therefore inform reimbursement and prioritization decisions [43].

These results are consistent with previous evidence regarding the clinical effectiveness of motor and sensory-based intensive interventions. Novak et al. have identified moderate to strong evidence for therapies such as Therasuit and structured physiotherapy [9, 10]. However, the economic evaluation of such therapies remains limited [11, 44], and many previous studies have focused on subjective or functional improvements e.g., through music therapy, hippotherapy, or acupuncture without embedding them in formal economic evaluation frameworks. This study addresses this gap by providing cost-utility estimates by therapy type, using validated instruments such as the EQ-5D-Y. From a health policy perspective, our findings support prioritizing high-value therapies like Therasuit and physiotherapy within pediatric CP management plans. The long-term model extended this analysis through a 30-year individual-level microsimulation enabling the estimation of cumulative health and cost outcomes, enhancing long-term resource allocation decisions. At the same time, the results highlight that although some non-standard rehabilitation interventions may offer added value in selected cases, their relatively high cost places them in a zone of economic uncertainty particularly when more conservative WTP thresholds are applied. Funding decisions should therefore not be based on the broad Intensive and Emerging Rehabilitation Therapies category, but rather on the individual performance of each therapy [14, 41, 43]. This underscores the need for personalized decision-making [14, 36, 37], guided by the clinical, functional, and socioeconomic characteristics of each child. Individualized approaches can better identify which patients are most likely to benefit from these therapies, thereby improving the efficiency of care and optimizing resource use [36, 38, 39]. Furthermore, a key methodological insight from this study concerns the discrepancy between short- and long-term cost-effectiveness estimates across therapies. This divergence is explained by accumulated effects that only emerge over extended time horizons. Intensive and emerging neurorehabilitation therapies typically lead to gradual clinical improvements—particularly in motor function and autonomy—that require sustained exposure to manifest fully. While these gains may appear modest in a 12-month follow-up, they may compound over time, leading to significant QALY improvements and delayed cost offsets, such as reduced hospitalizations, lower reliance on assistive devices, and decreased caregiving burden [15, 45].

The divergence between short- and long-term cost-effectiveness results should be interpreted within the context of the modelling framework rather than as a methodological inconsistency. The short-term analysis reflects immediate, observed responses, while the long-term microsimulation captures the dynamic evolution of cerebral palsy over time, incorporating heterogeneity in patient trajectories and persistence of therapeutic effects. This structure, which allows individual history to influence future transitions and outcomes, is particularly suited to chronic pediatric conditions, where gradual improvements and delayed cost offsets emerge beyond a one-year horizon. Therefore, the more favourable long-term ICERs likely reflect the realistic capture of sustained benefits rather than artefactual accumulation, though they remain subject to uncertainty in adherence, duration of effects, and progression parameters.

These dynamics are captured in our 30-year microsimulation model, which simulates individual transitions across GMFCS-defined health states and aggregates costs and QALYs accordingly. This approach is supported by evidence showing that cost-effectiveness outcomes can shift considerably when longer time horizons are used. For example, Kim et al. (2017) analysed 782 CEAs and found that among those reporting multiple time horizons, extending the horizon led to more favourable ICERs in 19 out of 23 cases. Their findings underscore that time horizon assumptions can substantially influence value assessments, and recommend using horizons long enough to capture all relevant consequences [45]. Similarly, Prosser et al. (2024), Basu and Maciejewski (2019), and Haacker et al. (2020) highlight the need for long-term modelling in pediatric and chronic care, not only to reflect delayed effects, but also to accommodate patient-level heterogeneity [46–48]. As noted by Drummond et al. (2015), the selected time horizon should align with the natural course and expected duration of intervention benefits [19]. Additionally, Shih et al. (2018) highlight the importance of capturing long-term consequences in economic evaluations of people with CP, taking into account, the characteristics of the condition [15].

This study has several *strengths*, including the use of comprehensive real-world data, a mixed-methods approach combining regression-based and simulation techniques, and adherence to the CHEERS 2022 reporting standards [26]. Nonetheless, certain *limitations* should be acknowledged. Non-random allocation to treatment groups may introduce selection bias, and although adjustments were made for covariates, residual confounding cannot be ruled out. Additionally, while the EQ-5D-Y is widely used and recommended for pediatric economic evaluations [12], its sensitivity in populations with severe disability may be limited. Indirect effects on caregivers or non-medical costs were also beyond the scope of this analysis. In addition, several therapy-specific analyses were based on very small subgroup sizes (e.g., *n* = 6 for homeopathy), which limits statistical precision and increases uncertainty around the corresponding cost-effectiveness estimates. Results for these therapies should therefore be interpreted with caution and considered exploratory rather than definitive.

From a methodological perspective, both the short- and long-term models present specific methodological limitations. The short-term SURE model, while grounded in observed data, may underestimate the persistence of therapeutic gains due to its limited follow-up and potential residual confounding from non-random treatment allocation. Conversely, the long-term microsimulation relies on assumptions regarding transition stability, adherence, and maintenance of effects that may not fully represent real-world variability. Although probabilistic sensitivity analyses address parameter uncertainty, structural uncertainty remains, as with most lifetime models in pediatric rehabilitation. Recognizing these factors enhances transparency and acknowledges where the models may fall short or yield results that are difficult to interpret.

Finally, one methodological *limitation* of our analysis is the exclusion of school holiday months (July–September) from the 12-month treatment window. While this decision improves the internal validity of treatment exposure and avoids diluting cost and effect estimates with periods of minimal intervention, it may lead to a slight underestimation of annual costs and limit generalizability to contexts where therapy is delivered year-round [30, 31, 49]. Additionally, families who continued some level of private therapy during these months may have incurred out-of-pocket costs not fully captured in our estimates. Nonetheless, the exclusion ensured consistent exposure assessment across both groups and improved the accuracy of cost-effectiveness comparisons. Future research should aim to validate these findings across broader and more diverse settings, assess the budgetary impact of integrating high-value therapies, and explore the inclusion of family and societal outcomes particularly relevant in chronic pediatric conditions such as CP. 

## Conclusions

This study provides a novel hybrid economic evaluation of Intensive and Emerging Rehabilitation Therapies (IERT) in paediatric cerebral palsy, combining real-world individual-level data with long-term microsimulation. At the strategic level (IERT + ST vs. ST), the model showed that supplementing standard care with intensive or emerging therapies can yield meaningful health gains and be cost-effective within commonly accepted Spanish willingness-to-pay (WTP) thresholds (€25,000–€60,000/QALY), especially when considering the long-term cumulative effects. At the therapy level, pre-specified short- and long-term analyses revealed substantial heterogeneity within the IERT group. Intensive physiotherapy and Therasuit were consistently cost-effective across both time horizons (≈ 92 % and 87 % probability at €60,000/QALY, respectively), whereas hippotherapy, speech therapy, and occupational therapy achieved cost-effectiveness only in the long term, due to accumulated benefits and sustained functional improvements that manifest over time, despite being dominated or exceeding the WTP threshold in the short term. These results underscore the importance of prioritizing highvalue interventions than treating all non-standard therapies as equally beneficial. Tailoring decisions to clinical profiles and socioeconomic contexts may improve the efficiency and equity of care. We acknowledge key limitations—regional observational cohort, modest sample sizes for some therapies, potential sensitivity constraints of EQ-5D-Y in severe disability, and a public-payer, direct-medical-cost perspective—which may affect precision and generalizability. Taken together, these findings should be viewed as a gateway to broader evaluations, motivating multicenter studies with larger samples and, where feasible, quasi-experimental or randomized designs; broader costing that includes caregiver and societal impacts; complementary preference-based HRQoL instruments alongside EQ-5D-Y; and budget-impact and equity-sensitive analyses to inform sustainable pediatric neurorehabilitation policy.

## Supplementary Information

Supplementary Material 1: Appendix A. The complete R code used for each of the decision microsimulation models is available in the GitHub repository associated with this study.

## Data Availability

Data from this study are available upon special and justified request through the lead author to the institution holding the data (Hospital Universitario de Navarra). This process is need because anonymized records for individual patients across more than one data source external to EPCINA cannot, and should not, be linked owing to a potential increase in risk of patient re-identification.
