# Supplementary material for: Cost-effectiveness analysis of intensive and emerging rehabilitation therapies in children with cerebral palsy: an observational cohort study using real-world evidence and microsimulation modelling
Source: Health Econ Rev. 2026 May 1;16:62. doi: 10.1186/s13561-026-00768-2 (PMC13177883; doi:10.1186/s13561-026-00768-2)
Supplement: Supplementary file 1 — Supplementary Material 1: Appendix A. The complete R code used for each of the decision microsimulation models is available in the GitHub repository associated with this study. [file 13561_2026_768_MOESM1_ESM.docx]

*Cost-Effectiveness Analysis of Intensive and Emerging Rehabilitation Therapies in Children with Cerebral Palsy: Real-World Evidence and Microsimulation Modelling*

**Supplementary material**

**Table S1.** Unit costs (in 2023 euros), annual quantities (mean per child), and mean annual costs for Standard Treatment and Intensive and Emerging Rehabilitation Therapies (IERT). Quantities for IERT items represent averages across IERT participants (n=92); public healthcare items correspond to the whole sample (n=148) unless otherwise specified

| **Resource** | **Unit Cost (€2023)** | **Source Unit Cost** | **Quantity  per year  (child mean)** | **Annual Cost Mean (€2023)** |
| --- | --- | --- | --- | --- |
| **Emerging and Intensive Rehabilitation Therapies n = 92** |  |  |  | **5683**ᵃ |
| ***Emerging Rehabilitation Therapies (per session) n=87*** |  |  |  | **4179**ᵇ |
| Hippotherapy | 74 | 1,3,4 | 61 | 4514 |
| Therasuit | 63 | 1,3,4 | 72 | 4536 |
| Peto Method | 49 | 1,3,4 | 87 | 4263 |
| Homeopathy | 49 | 1,3,4 | 73 | 3577 |
| ***Intensive Rehabilitation Therapies (per session) n= 92*** |  |  |  | **7187**ᵇ |
| Extra physiotherapy/rehabilitation sessions | 75 | 1,3,4 | 100 | 7500 |
| Extra speech therapy sessions | 80 | 1,3,4 | 90 | 7200 |
| Extra occupational therapy sessions | 74 | 1,3,4 | 88 | 6500 |
| **Health care costs: Standard Treatment n = 148** |  |  |  | **5396**ᶜ |
| General practitioner care (per visit) | 47 |  | 1 | 47 |
| *Specialist medical care (per visit)* |  |  |  |  |
| Neurologist | 200 | 1,2,4 | 1,5 | 300 |
| Pediatrician | 150 | 1,2,4 | 1,5 | 225 |
| Rehabilitation specialist | 85 | 1,2,4 | 1,62 | 138 |
| Orthopedist | 70 | 1,2,4 | 1,5 | 105 |
| Ear, nose and throat specialist | 45 | 1,2,4 | 1 | 45 |
| ***Diagnostic Test (per test)*** |  |  |  |  |
| X-ray hip joints | 65 | 1,2,4,5 | 0,46 | 30 |
| X-ray spinal column | 300 | 1,2,4,5 | 0,08 | 24 |
| MRI-spinal column | 250 | 1,2,4,5 | 0,16 | 40 |
| MRI-knee | 75 | 1,2,4,5 | 0,33 | 25 |
| Electroencephalogram | 150 | 1,2,4,5 | 0,23 | 35 |
| Barium swallowing test | 100 | 1,2,4,5 | 0,35 | 35 |
| Salivary measurement | 200 | 1,2,4,5 | 0,15 | 30 |
| Hospitalisation (per day) | 500 | 1.2,4,5 | 1 | 500 |
| *Medication (per dose/visit)* |  |  |  |  |
| Botulinum toxin treatment | 450 | 2,5 | 1 | 450 |
| CP and comorbidities | 250 | 2,5 | 4,04 | 1010,6 |
| *Standard Therapies (per session)* |  |  |  |  |
| Rehabilitation | 30 | 1,2,4 | 64 | 1920 |
| Speech therapy | 25 | 1,2,4 | 32 | 800 |
| Occupational therapy | 25,58 | 1,2,4 | 22 | 562,8 |
| **Total Healthcare cost IERT + Standard Treatment (€)** | - |  | - | **11,079** |
| Sources: (1) Spanish guideline for PharmacoEconomics research, (2) Data University Hospital Navarra, (3) Out-of-pocket expenses in private centers, (4) Public rates BON, (5) Pharmacy and Benefits Service of the Navarra Health Service. *All data from these sources were collected through the cerebral palsy burden questionnaire (EBQ-CP).  All costs are expressed in 2023 euros (€) and represent mean annual values per child. Unit costs refer to cost per session, visit, or day, as applicable, and annual quantities indicate the average yearly number of uses per child.  **IERT:** Intensive and Emerging Rehabilitation Therapies. This group includes two partially overlapping subgroups: **ERT** (Emerging Rehabilitation Therapies: hippotherapy, Petö Method, Therasuit, homeopathy) and **IRT** (Intensive Rehabilitation Therapies: physiotherapy, speech therapy, occupational therapy). Within the IERT group, patients receive therapies from both subgroups, but no therapy is double counted: each therapy type is recorded once per patient. Therefore, **subgroup totals (ERT and IRT) are descriptive and not additive**, ensuring that **the overall IERT cost reflects unique, non-duplicated expenditures**.  ᵃ Overall average annual cost of IERT, based on estimated partial averages of costs per subgroup: (4179+7187)/2 = 5683 or the overall average estimate for all therapies  ᵇ Partial annual average cost, based on estimated averages for therapies at the IRT or ERT subgroup level  ᶜ Overall average cost of standard treatment for all patients (comparator group: 5,156 + intervention group: 5,636) / 2 = 5,396 | | | | |

**Table 2b.** Annual treatment costs (€2023), public healthcare system perspective. Main comparison: standard treatment versus IERT + standard treatment; exploratory subgroups (ERT/IRT).

| Resource Category | Mean annual cost (€) | | |
| --- | --- | --- | --- |
|  | IERT + Standard Treatment *Primary Intervention* (n = 92) | | Standard Treatment *Primary Comparator* (n = 56) |
|  | ERTᵃ +ST (n = 87*) | IRTᵇ+ST (n = 92) |  |
| **Intensive and Emerging Rehabilitation therapies (IERT)** | **4179** | **7187** | **0** |
| **Health care costs (excluding IERT)** | **5396** | **5633** | **5156** |
| General practitioner care | 46 | 46 | 47 |
| Specialist medical care | 202 | 201 | 196 |
| Diagnostic Tests | 219 | 218 | 222 |
| Hospitalisation | 735 | 735 | 643 |
| Medication | 1082 | 1082 | 878 |
| Standard Therapies | 3352 | 3351 | 3170 |
| **Total annual healthcare costs** | **9815** | **12820** | **5156** |
| **Base-case ΔCost (IERT+ST − Standard) ᶜ** | *4659* | *7664* |  |

**Note.** Data are means (2023 €). **n** denotes non-missing observations; column denominators are shown in the headers. **IERT** = Intensive and Emerging Rehabilitation Therapies. **ᵃERT** = Emerging Rehabilitation Therapies (hippotherapy, Petö Method, Therasuit, homeopathy); **ᵇIRT** = Intensive Rehabilitation Therapies (physiotherapy, speech therapy, occupational therapy). ERT and IRT are overlapping subgroups within IERT; therefore, columns are not additive**.** **ᶜ** **Base-case ΔCost (IERT+ST − Standard)** = difference in Total annual healthcare costs versus Standard treatment (e.g., 9,815 – 5,156 = 4,659 for ERT; 12,820 – 5,156 = 7,664 for IRT). Base-case cost-effectiveness (ΔQALYs and ICER) for IERT+ST vs Standard is reported in Table 3 (12-month SURE). * Five participants in the ERT subgroup were lost to follow-up; ERT cost analysis uses n = 87 complete cases (see flow diagram).

**Table S2**. General input parameters of the illustrative microsimulation model in a paediatric cerebral palsy population

| **Parameter Description** | **R Name** | **Value** | **Notes** |
| --- | --- | --- | --- |
| **General Settings** |  |  |  |
| Time horizon | n.t | 30 y | Years |
| Cycle length | cl | 1 y | Annual |
| Number of simulated individuals | n.i | 100.000 | – |
| Names of health states | v.n | H, S1, S2, D | GMFCS I-II (walks without limitations: H), GMFCS III  (walks with limitations or uses assistive devices: S1), GMFCS IV-V (non-ambulatory or uses a wheelchair: S2), Dead (D) |
| **Discounting** |  |  | – |
| Annual discount rate (costs/QALYs) | d.c/d.e | 3% | – |
| **Annual Transition Probabilities** |  |  | – |
| H → S1 (Disease onset) | p.HS1 | 0.08 | Annual (1) (2) |
| S1 → H (Recovery) | p.S1H | 0.015 | Annual (1) |
| S1 → S2 (Progression) | p.S1S2 | 0.05 | Annual (1) |
| **Annual risks of death** |  |  | – |
| H → D (baseline mortality) | p.HD | 0.002 | Annual (1) |
| Rate ratio of death in S1 vs. H | rr.S1 | 3 | (1) |
| Rate ratio of death in S2 vs. H | rr.S2 | 10 | (1) |
| **Annual costs** |  |  |  |
| GMFCS I-II individuals | c.H | $2,000 | (2) |
| GMFCS III individuals in S1 | c.S1 | $4,000 | (2) |
| GMFCS IV-V individuals in S2 | c.S2 | $15,000 | (2) |
| Annual Standard Treatment cost (S1 & S2) | c.Trt | $5156 | (2) |
| Annual Intensive Rehabilitation Therapies cost (S1 & S2). * | c.TC | $12820 | (2) |
| Annual Emerging Rehabilitation therapies cost (S1 & S2). * | c.TAL | $9815 | (2) |
| **Utility weights** |  |  |  |
| GMFCS I-II individuals | u.H | 0,9 | (2) |
| GMFCS III individuals in S1 | u.S1 | 0,65 | (2) |
| GMFCS IV-V individuals in S2 | u.S2 | 0,5 | (2) |
| **Intervention effect** |  |  | – |
| Utility when GMFCS III(IV-V) and being  treated with standard treatment | u.Trt | 0,75 | (2) |
| Utility when GMFCS III(IV-V) and being  treated with Intensive Rehab therapies* | u.TC | 0,85 | (2) |
| Utility when GMFCS III(IV-V) and being  treated with Emerging Rehab therapies* | u.TAL | 0,8 | (2) |

**Notes:** All parameter values correspond to annual estimates expressed in 2023 euros (€) and were used in the base-case microsimulation model over a 30-year time horizon. Transition probabilities are applied per cycle (1 year). Costs refer to average annual direct medical costs per child, stratified by GMFCS level. Utility weights represent mean EQ-5D-Y–based values for each health state, adjusted by treatment type. Intervention effects reflect incremental improvements in utility associated with therapy use.

(1) Data source: Published literature (see references below).
(2) Data source: Real-world data from our observational cohort.
All model parameters were parameterized and implemented in R following the methodological framework described by Krijkamp et al. (2018) for first-order individual-level microsimulation models.
*As a group of therapies or as individual therapy. It has been stratified by GMFCS groups for each therapy in Appendices A.1 and A.2.*

**Published Literature:**

**Fuente 1) Annual transitions probabilities and Annual risk of death**

- Amankwah, N., Oskoui, M., Garner, R., Bancej, C., Manuel, D.G., Wall, R., Finès, P., Bernier, J., Tu, K., Reimer, K.: Cerebral palsy in Canada, 2011-2031: results of a microsimulation modelling study of epidemiological and cost impacts. Health Promot. Chronic Dis. Prev. Can. Res. Policy Pract. 40, 25–37 (2020). <https://doi.org/10.24095/hpcdp.40.2.01>
- Krijkamp, E.M., Alarid-Escudero, F., Enns, E.A., Jalal, H.J., Hunink, M.G.M., Pechlivanoglou, P.: Microsimulation Modeling for Health Decision Sciences Using R: A Tutorial. Med. Decis. Making. 38, 400–422 (2018). <https://doi.org/10.1177/0272989X18754513>
- Zucchelli, E., Jones, A.M., Rice, N.: The evaluation of health policies through microsimulation methods. Health Econom. Data Group HEDG Work. Pap. (2010)
- Shih, S.T.F., Tonmukayakul, U., Imms, C., Reddihough, D., Graham, H.K., Cox, L., Carter, R.: Economic evaluation and cost of interventions for cerebral palsy: a systematic review. Dev. Med. Child Neurol. 60, 543–558 (2018). <https://doi.org/10.1111/dmcn.13653>
- Arnaud, C., Ehlinger, V., Perraud, A., Kinsner-Ovaskainen, A., Klapouszczak, D., Himmelmann, K., Petra, M., Rackauskaite, G., Lanzoni, M., Platt, M.-J., Delobel-Ayoub, M.: Public health indicators for cerebral palsy: A European collaborative study of the Surveillance of Cerebral Palsy in Europe network. Paediatr. Perinat. Epidemiol. 37, 404–412 (2023). <https://doi.org/10.1111/ppe.12950>
- Goldsmith, S., McIntyre, S., Blair, E., Smithers-Sheedy, H., Badawi, N., Hansen, M.: Cerebral Palsy: Epidemiology. In: Eisenstat, D.D., Goldowitz, D., Oberlander, T.F., and Yager, J.Y. (eds.) Neurodevelopmental Pediatrics: Genetic and Environmental Influences. pp. 479–495. Springer International Publishing, Cham (2023)
- Prosser, L.A., Pierce, S.R., Skorup, J.A., Paremski, A.C., Alcott, M., Bochnak, M., Ruwaih, N., Jawad, A.F.: Motor training for young children with cerebral palsy: A single-blind randomized controlled trial. Dev. Med. Child Neurol. 66, 233–243 (2024). <https://doi.org/10.1111/dmcn.15729>
- Basu, A., Maciejewski, M.L.: Choosing a Time Horizon in Cost and Cost-effectiveness Analyses. JAMA. 321, 1096–1097 (2019). https://doi.org/10.1001/jama.2019.1153

**Fuente 2) Annual Costs**

- Data source: Our Real-world Data from our observational cohort
- Nova Díaz, D.M., Arana Rivera, P., Sánchez Iriso, E., Aguilera-Albesa, S.: The Economic and Social Burden of Pediatric Cerebral Palsy in Spain: A Cost-of-Illness Study. Front. Public Health. 13, (2025). <https://doi.org/10.3389/fpubh.2025.1589114>

**Appendix A**

**OPEN-SOURCE REPOSITORY**

GitHub repository with R code of the microsimulation model:

Appendix A: ([MICROSIM_PHE.R](https://github.com/Diana-MND1996/microsimulation-cerebralpalsy/blob/main/MICROSIM_PHE.R)) Microsimulation model structure and parameters. Cost-effectiveness analysis of Intensive Rehabilitation Therapies, Emerging Rehabilitation Therapies, standard treatment versus no treatment.

Appendix A.1: ([Microsim_EmergingRehabTherapies.R](https://github.com/Diana-MND1996/microsimulation-cerebralpalsy/blob/main/Microsim_EmergingRehabTherapies.R):) Cost-effectiveness analysis of each of the Emerging Rehabilitation Therapies versus standard treatment.

Appendix A.2: ([Microsim_IntensiveRehabTherapies.R](https://github.com/Diana-MND1996/microsimulation-cerebralpalsy/blob/main/Microsim_IntensiveRehabTherapies.R)) Cost-effectiveness analysis of each of Intensive Rehabilitation Therapies versus standard treatment.

All the code together can be found in this README file:

<https://github.com/Diana-MND1996/microsimulation-cerebralpalsy/blob/main/README.md>

# CHEERS 2022 Checklist

**Table S3.** Completed CHEERS 2022 Checklist

| **Topic** | **No.** | **Item** | **Location where item is reported** |
| --- | --- | --- | --- |
| **Title** |  |  |  |
|  | 1 | Identify the study as an economic evaluation and specify the interventions being compared. | Title, Page 1 |
| **Abstract** |  |  |  |
|  | 2 | Provide a structured summary that highlights context, key methods, results, and alternative analyses. | Abstract, Page 1 |
| **Introduction** |  |  |  |
| **Background and objectives** | 3 | Give the context for the study, the study question, and its practical relevance for decision making in policy or practice. | Introduction, Page 2-3 |
| **Methods** |  |  |  |
| **Health economic analysis plan** | 4 | Indicate whether a health economic analysis plan was developed and where available. | Not reported |
| **Study population** | 5 | Describe characteristics of the study population (such as age range, demographics, socioeconomic, or clinical characteristics). | Methods, Subsection 2.2 |
| **Setting and location** | 6 | Provide relevant contextual information that may influence findings. | Methods, Subsection 2.1 and 2.3 |
| **Comparators** | 7 | Describe the interventions or strategies being compared and why chosen. | Methods, Subsection 2.4 |
| **Perspective** | 8 | State the perspective(s) adopted by the study and why chosen. | Methods, Subsection 2.1 |
| **Time horizon** | 9 | State the time horizon for the study and why appropriate. | Methods, Subsection 2.1 |
| **Discount rate** | 10 | Report the discount rate(s) and reason chosen. | Methods, Subsection 2.3 |
| **Selection of outcomes** | 11 | Describe what outcomes were used as the measure(s) of benefit(s) and harm(s). | Methods, Subsection 2.5 |
| **Measurement of outcomes** | 12 | Describe how outcomes used to capture benefit(s) and harm(s) were measured. | Methods, Subsection 2.5 |
| **Valuation of outcomes** | 13 | Describe the population and methods used to measure and value outcomes. | Methods, Subsection 2.5 |
| **Measurement and valuation of resources and costs** | 14 | Describe how costs were valued. | Methods, Subsection 2.3 |
| **Currency, price date, and conversion** | 15 | Report the dates of the estimated resource quantities and unit costs, plus the currency and year of conversion. | Methods, Subsection 2.3 |
| **Rationale and description of model** | 16 | If modelling is used, describe in detail and why used. Report if the model is publicly available and where it can be accessed. | Methods, Subsection 2.6 and supplementary material |
| **Analytics and assumptions** | 17 | Describe any methods for analysing or statistically transforming data, any extrapolation methods, and approaches for validating any model used. | Methods, Subsection 2.6.1 and 2.6.2 |
| **Characterizing heterogeneity** | 18 | Describe any methods used for estimating how the results of the study vary for subgroups. | Methods, Subsection 2.6.2 |
| **Characterizing distributional effects** | 19 | Describe how impacts are distributed across different individuals or adjustments made to reflect priority populations. | Not Applicable |
| **Characterizing uncertainty** | 20 | Describe methods to characterize any sources of uncertainty in the analysis. | Methods, Subsection 2.7 |
| **Approach to engagement with patients and others affected by the study** | 21 | Describe any approaches to engage patients or service recipients, the general public, communities, or stakeholders (such as clinicians or payers) in the design of the study. | Declarations section |
| **Results** |  |  |  |
| **Study parameters** | 22 | Report all analytic inputs (such as values, ranges, references) including uncertainty or distributional assumptions. | Results, Page 8-9. |
| **Summary of main results** | 23 | Report the mean values for the main categories of costs and outcomes of interest and summarize them in the most appropriate overall measure. | Results, subsection 3.1, and Supplementary material Table S1. Subsection 3.2 and 3.3. |
| **Effect of uncertainty** | 24 | Describe how uncertainty about analytic judgments, inputs, or projections affect findings. Report the effect of choice of discount rate and time horizon, if applicable. | Results, Subsection 3.4 |
| **Effect of engagement with patients and others affected by the study** | 25 | Report on any difference patient/service recipient, general public, community, or stakeholder involvement made to the approach or findings of the study | Not applicable |
| **Discussion** |  |  |  |
| **Study findings, limitations, generalizability, and current knowledge** | 26 | Report key findings, limitations, ethical or equity considerations not captured, and how these could affect patients, policy, or practice. | Discussion, Page 15. |
| **Other relevant information** |  |  |  |
| **Source of funding** | 27 | Describe how the study was funded and any role of the funder in the identification, design, conduct, and reporting of the analysis | End of manuscript or Declarations statements section |
| **Conflicts of interest** | 28 | Report authors conflicts of interest according to journal or International Committee of Medical Journal Editors requirements. | End of manuscript or Declarations statements section |

*From:* Husereau D, Drummond M, Augustovski F, et al. Consolidated Health Economic Evaluation Reporting Standards 2022 (CHEERS 2022) Explanation and Elaboration: A Report of the ISPOR CHEERS II Good Practices Task Force. Value Health 2022;25. <doi:10.1016/j.jval.2021.10.008>

**Table S4.** STROBE Statement—Checklist of items that should be included in reports of ***cohort studies***

|  | **Item No** | **Recommendation** |
| --- | --- | --- |
| **Title and abstract** | 1 | (*a*) Indicate the study’s design with a commonly used term in the title or the abstract |
|  |  | (*b*) Provide in the abstract an informative and balanced summary of what was done and what was found |
| **Introduction** | | |
| Background/rationale | 2 | Explain the scientific background and rationale for the investigation being reported |
| Objectives | 3 | State specific objectives, including any prespecified hypotheses |
| **Methods** | | |
| Study design | 4 | Present key elements of study design early in the paper |
| Setting | 5 | Describe the setting, locations, and relevant dates, including periods of recruitment, exposure, follow-up, and data collection |
| Participants | 6 | (*a*) Give the eligibility criteria, and the sources and methods of selection of participants. Describe methods of follow-up |
|  |  | (*b*) For matched studies, give matching criteria and number of exposed and unexposed |
| Variables | 7 | Clearly define all outcomes, exposures, predictors, potential confounders, and effect modifiers. Give diagnostic criteria, if applicable |
| Data sources/ measurement | 8* | For each variable of interest, give sources of data and details of methods of assessment (measurement). Describe comparability of assessment methods if there is more than one group |
| Bias | 9 | Describe any efforts to address potential sources of bias |
| Study size | 10 | Explain how the study size was arrived at |
| Quantitative variables | 11 | Explain how quantitative variables were handled in the analyses. If applicable, describe which groupings were chosen and why |
| Statistical methods | 12 | (*a*) Describe all statistical methods, including those used to control for confounding |
|  |  | (*b*) Describe any methods used to examine subgroups and interactions |
|  |  | (*c*) Explain how missing data were addressed |
|  |  | (*d*) If applicable, explain how loss to follow-up was addressed |
|  |  | (*e*) Describe any sensitivity analyses |
| **Results** | | |
| Participants | 13* | (a) Report numbers of individuals at each stage of study—eg numbers potentially eligible, examined for eligibility, confirmed eligible, included in the study, completing follow-up, and analysed |
|  |  | (b) Give reasons for non-participation at each stage |
|  |  | (c) Consider use of a flow diagram |
| Descriptive data | 14* | (a) Give characteristics of study participants (eg demographic, clinical, social) and information on exposures and potential confounders |
|  |  | (b) Indicate number of participants with missing data for each variable of interest |
|  |  | (c) Summarise follow-up time (eg, average and total amount) |
| Outcome data | 15* | Report numbers of outcome events or summary measures over time |
| Main results | 16 | (*a*) Give unadjusted estimates and, if applicable, confounder-adjusted estimates and their precision (eg, 95% confidence interval). Make clear which confounders were adjusted for and why they were included |
|  |  | (*b*) Report category boundaries when continuous variables were categorized |
|  |  | (*c*) If relevant, consider translating estimates of relative risk into absolute risk for a meaningful time period |
| Other analyses | 17 | Report other analyses done—eg analyses of subgroups and interactions, and sensitivity analyses |
| **Discussion** | | |
| Key results | 18 | Summarise key results with reference to study objectives |
| Limitations | 19 | Discuss limitations of the study, taking into account sources of potential bias or imprecision. Discuss both direction and magnitude of any potential bias |
| Interpretation | 20 | Give a cautious overall interpretation of results considering objectives, limitations, multiplicity of analyses, results from similar studies, and other relevant evidence |
| Generalisability | 21 | Discuss the generalisability (external validity) of the study results |
| **Other information** | | |
| Funding | 22 | Give the source of funding and the role of the funders for the present study and, if applicable, for the original study on which the present article is based |

*Give information separately for exposed and unexposed groups.

**Note:** An Explanation and Elaboration article discusses each checklist item and gives methodological background and published examples of transparent reporting. The STROBE checklist is best used in conjunction with this article (freely available on the Web sites of PLoS Medicine at http://www.plosmedicine.org/, Annals of Internal Medicine at http://www.annals.org/, and Epidemiology at http://www.epidem.com/). Information on the STROBE Initiative is available at http://www.strobe-statement.org.
